# Exacerbation of Elastase-Induced Emphysema via Increased Oxidative Stress in Metallothionein-Knockout Mice

**DOI:** 10.3390/biom12040583

**Published:** 2022-04-15

**Authors:** Ken Ichiro Tanaka, Sachie Shiota, Okina Sakakibara, Mikako Shimoda, Ayaka Takafuji, Misaki Takabatake, Yoshito Kadota, Takashige Kawakami, Shinya Suzuki, Masahiro Kawahara

**Affiliations:** 1 Laboratory of Bio-Analytical Chemistry, Research Institute of Pharmaceutical Sciences, Faculty of Pharmacy, Musashino University, 1-1-20 Shinmachi, Nishitokyo 202-8585, Japan; s1743142@stu.musashino-u.ac.jp (S.S.); s1743037@stu.musashino-u.ac.jp (O.S.); s1343142@stu.musashino-u.ac.jp (M.S.); s1243088@stu.musashino-u.ac.jp (A.T.); s1443123@stu.musashino-u.ac.jp (M.T.); makawa@musashino-u.ac.jp (M.K.); 2 Faculty of Pharmaceutical Sciences, Tokushima Bunri University, Tokushima 770-8514, Japan; ykadota@ph.bunri-u.ac.jp (Y.K.); tkawakami@ph.bunri-u.ac.jp (T.K.); suzukis@ph.bunri-u.ac.jp (S.S.)

**Keywords:** COPD, oxidative stress, metallothionein, ROS, in vivo imaging system, elastase

## Abstract

Although the pathogenesis of chronic obstructive pulmonary disease (COPD) is not yet fully understood, recent studies suggest that the disruption of the intracellular balance of oxidative (such as reactive oxygen species (ROS)) and antioxidant molecules plays an important role in COPD development and progression. Metallothionein is an endogenous metal-binding protein with reported ROS scavenging activity. Although there have been many publications on the protective effects of metallothionein in the kidney and liver, its role in COPD models such as elastase- or cigarette smoke (CS)-induced lung injury is unknown. Thus, in the present study, we analyzed the elastase-induced lung injury model using metallothionein-knockout (MT-KO; MT-1 and -2 gene deletion) mice. The expression of MT-1 and MT-2 in the lungs of MT-KO mice was markedly lower compared with that in the lungs of wildtype (WT) mice. Porcine pancreatic elastase (PPE)-induced lung injury (alveolar enlargement and respiratory impairment) was significantly exacerbated in MT-KO mice compared with WT mice. Additionally, PPE-induced increases in the number of inflammatory cells, inflammatory cytokines, and cell death in lung tissue were significantly more pronounced in MT-KO mice compared with WT mice. Finally, using an in vivo imaging system, we also found that PPE-induced ROS production in the lungs was enhanced in MT-KO mice compared with WT mice. These results suggest that metallothionein may act as an inhibitor against elastase-induced lung injury by suppressing ROS production. These results suggest that metallothionein protein, or compounds that can induce metallothionein, could be useful in the treatment of COPD.

## 1. Introduction

Chronic obstructive pulmonary disease (COPD) is a serious health problem in which progressive and irreversible airflow limitations are observed, and it is caused by an abnormal inflammatory response to cigarette smoke (CS) and air pollution. Long-acting β_2_-agonists (LABAs) and muscarinic receptor antagonists (LAMAs) are used clinically for bronchodilation in the treatment of COPD [1,2]. Although steroid therapy is commonly used to treat inflammation associated with COPD, there are some cases in which steroid therapy does not sufficiently suppress COPD disease progression because of resistance to steroid therapy in part of the inflammatory processes [1,3]. Therefore, it is important to discover new proteins that inhibit the onset and exacerbation of COPD and to develop new types of anti-inflammatory compounds for treating COPD.

While the pathogenesis of COPD is not yet fully understood, recent studies suggest that the cellular redox state, determined by the balance between oxidative molecules such as reactive oxygen species (ROS) and antioxidant molecules, plays an important role in this process [4]. In fact, it has been confirmed that oxidative stress is higher in the bronchoalveolar lavage fluid (BALF), sputum, and exhaled breath condensate of COPD patients compared with healthy subjects [5,6]. Moreover, in addition to the fact that CS contains high concentrations of ROS, leukocytes, particularly neutrophils, activated by CS produce excess ROS, such as superoxide anion, via nicotinamide adenine dinucleotide phosphate (NADPH) oxidase [7,8]. Thus, antioxidant molecules have attracted attention as candidates for COPD treatment, and their effects have been confirmed in COPD animal models induced by elastase or CS [9,10].

Metallothioneins are intracellular cysteine-rich proteins that maintain metal homeostasis. They were first isolated from horse kidney as cadmium-binding proteins in 1957. To date, four metallothionein isoforms (MT-1, -2, -3, and -4) have been identified in mammals. Among these metallothionein isoforms, MT-1 and MT-2 in particular have been found to be expressed in most organs [11]. Metallothionein can be induced not only by heavy metals, such as cadmium, but also by less biologically toxic metals, such as zinc (Zn) [12]. In addition to detoxifying heavy metals such as cadmium by binding to them, metallothionein also exerts antioxidant effects by binding ROS to their own thiol groups, thus exerting protective effects in disease models in various organs [13]. Many studies have shown a protective role for metallothionein in the kidney and liver; for example, studies using metallothionein-knockout (MT-KO) mice have shown that metallothioneins have protective effects against hypoxia-induced renal injury and drug-induced liver injury [14,15,16]. In contrast, there are few reports describing metallothionein protection in the lungs. Specifically, previous studies using MT-KO mice showed that metallothionein inhibits acute lung injury caused by paraquat or nickel, but similar protection has not yet been investigated in a COPD model [17,18]. We speculate that metallothionein proteins, or compounds that can induce metallothioneins, may be useful in the treatment of COPD.

Polaprezinc, which is used clinically as an anti-ulcer drug, is a chelate compound consisting of carnosine and Zn [19]. We previously conducted experiments with the hope that polaprezinc would exert a cytoprotective effect by inducing metallothionein proteins. As a result, we found that polaprezinc clearly inhibits lung epithelial cell death induced by cadmium, a toxic metal contained in CS, by suppressing ROS production via metallothionein induction [20]. However, no studies to analyze the efficacy of metallothionein in the COPD models of porcine pancreatic elastase (PPE)-induced or CS-induced lung injury have been conducted using genetically modified mice. Here, we analyzed PPE-induced lung injury in MT-KO mice and their WT littermates. Furthermore, we also analyzed the PPE-induced ROS production in both types of mice using an in vivo imaging system.

## 2. Materials and Methods

### 2.1. Chemicals and Animals

Diff-Quik staining solution was purchased from Sysmex (Kobe, Japan). Luminal-based chemiluminescent probe (L-012) and isoflurane were obtained from Fujifilm Wako Pure Chemical Corporation (Tokyo, Japan). Novo-Heparin (5000 units), suitable for injection, was purchased from Mochida Pharmaceutical (Tokyo, Japan). The RNeasy^®^ kit was obtained from Qiagen (Hilden, Germany), PrimeScript™ II 1st strand cDNA Synthesis kit was obtained from Takara Bio (Shiga, Japan), and THUNDERBIRD^®^ NEXT SYBR qPCR Mix was obtained from Toyobo (Osaka, Japan). MT-KO mice (129/Sv-MT1MT2*^tm1Bri^*) developed by Masters et al. [21] and 129/SvCPJ (WT) mice were purchased from Jackson Laboratory (Bar Harbor, ME, USA). To maintain the same genetic background, only mice of the 129/SvCPJ strain were used for mating. Mice were housed in a controlled room (temperature, 22 °C; humidity, 55% ± 5%) under a 12-h light/dark cycle, fed a controlled diet (MF, Oriental Yeast Co., Ltd., Tokyo, Japan), and given ad libitum water. Mice aged 8–10 weeks were used in the experiments. The sex of the mice did not correlate with the extent of PPE-induced lung injury (Supplementary Figure S1). Since the number of mice born is not large, both male and female mice were used in this study. The experiments and procedures described here were carried out in accordance with the Guide for the Care and Use of Laboratory Animals as adopted and promulgated by the National Institutes of Health (Bethesda, MD, USA) and were approved by the Animal Care Committee of Musashino University.

### 2.2. Preparation of BALF

BALF was collected by cannulating the trachea and lavaging the lung twice with 1 mL of sterile saline containing 50 units/mL heparin. Approximately 1.8 mL of BALF was routinely recovered from each mouse, and the total cell number in the BALF was counted using a hemocytometer. After centrifugation with a Cytospin4^®^ (Thermo Fisher Scientific, Waltham, MA, USA), the cells were stained with Diff-Quik reagents, and the ratio of neutrophils or macrophages to the total cell number was determined. The amount of protein present in the BALF was evaluated by using the Bradford method (Takara Bio).

### 2.3. Measurement of ROS by In Vivo Imaging Analysis

In vivo imaging of ROS in mice was performed as previously described [22,23]. We used the FUSION chemiluminescence imaging system (Vilber Lourmat, Collégien, France). The ROS-sensing chemiluminescent probe, L-012, in sterile saline (75 mg/kg) was intraperitoneally administered to mice 24 h after the administration of PPE. The mice were euthanized 15 min after the L-012 administration, and their lungs were promptly dissected and imaged (5-min exposure). All data were analyzed using the FUSION chemiluminescence imaging system software.

### 2.4. Histological and Immunohistochemical Analyses

Lung tissue samples were fixed in 10% formalin neutral buffer solution for 24 h at a pressure of 25 cm H_2_O and then embedded in paraffin before being cut into 4-µm-thick sections. For histological examination, sections were stained first with Mayer’s hematoxylin and then with 1% eosin alcohol solution (H&E staining). Samples were mounted with malinol and inspected with the aid of an Olympus BX51 microscope (Tokyo, Japan).

To determine the mean linear intercept (MLI; an indicator of airspace enlargement), 20 lines (800 µm) were drawn randomly on the image of an H&E-stained section, and the intersection points with the alveolar walls were counted. For example, if there are 10 intersection points with the alveolar walls on an 800-µm line, the MLI = 80 µm. Twenty MLI points were measured for each tissue section, and the mean values are shown in figure The morphometric analysis at the light microscopic level was conducted by an investigator blinded to the study protocol.

For the immunohistochemical analysis of 8-OHdG, sections were first incubated with proteinase K (20 µg/mL) for 15 min at 37 °C, then with 0.3% hydrogen peroxide for 5 min, and finally with 2.5% goat serum for 10 min. They were then incubated with an antibody against 8-OHdG (1:200 dilution) for 12 h, followed by an incubation with a peroxidase-labeled polymer conjugated to goat anti-mouse immunoglobulin for 1 h. After 3,3′-diaminobenzidine was applied to the sections, they were incubated with Mayer’s hematoxylin. Finally, the sections were mounted in malinol and examined using a microscope. Image J software was used to calculate the 8-OHdG-positive area as a percentage of the total.

For terminal deoxynucleotidyl transferase biotin-dUTP nick end labeling (TUNEL) assays, sections were first incubated with proteinase K (20 µg/mL) for 15 min at 37 °C, then with terminal deoxynucleotidyl transferase and biotin 14-ATP for 1 h at 37 °C, and finally with Alexa Fluor 488 conjugated to streptavidin and 4′,6-diamidino-2-phenylindole (DAPI; 5 µg/mL) for 2 h. The sections then were mounted in Vectashield and examined using a fluorescence microscope. Image J software was used to count the number of TUNEL-positive cells.

### 2.5. Real-Time Reverse Transcription (RT) Polymerase Chain Reaction (PCR) Analysis

Total RNA was extracted from lung tissue using an RNeasy kit in accordance with the manufacturer’s protocol. Samples were reverse-transcribed using the PrimeScript™ II 1st strand cDNA Synthesis kit. The synthesized cDNA was used in real-time PCR experiments with THUNDERBIRD^®^ Next SYBR qPCR Mix and analyzed with a Bio-Rad (Hercules, CA, USA) CFX96™ real-time system and CFX Manager™ software. Specificity was confirmed by an electrophoretic analysis of the reaction products and the inclusion of template- or reverse transcriptase-free controls. To normalize the amount of total RNA present in each reaction, glyceraldehyde-3-phosphate dehydrogenase (Gapdh) cDNA was used as an internal standard. Primers were designed using the Primer-BLAST website (https://www.ncbi.nlm.nih.gov/tools/primer-blast/, accessed on 25 January 2022). Primer sequences will be provided upon request.

### 2.6. Treatment of Mice with PPE

After anesthetization with isoflurane, mice were intratracheally administered PPE (10 U/kg) in sterile saline using a P200 micropipette. During PPE administration, the mouse’s nostrils were blocked so that the solutions were inhaled from the mouth into the respiratory tract as the animal breathed. The control group received sterile saline intratracheally.

### 2.7. Analyses of Lung Mechanics and Respiratory Functions

The measurement of lung mechanics was performed with a computer-controlled small-animal ventilator (FlexiVent, SCIREQ, Montreal, QC, Canada) [24,25]. After mice were anesthetized with a triple anesthetic combination (0.75 mg/kg medetomidine, 4 mg/kg midazolam, and 5 mg/kg butorphanol), a tracheotomy was performed, and an 8-mm section of metallic tube was inserted into the trachea. Mice were then mechanically ventilated at a rate of 150 breaths/min, using a tidal volume of 8.7 mL/kg and a positive end-expiratory pressure of 2–3 cm H_2_O. Total lung capacity (TLC), total respiratory system elastance, tissue elastance and tissue damping were determined using FlexiVent software (version 5.3; SCIREQ, Montreal, QC, Canada).

Determination of the forced expiratory volume in 0.05 s (FEV0.05)/forced vital capacity (FVC) or forced expiratory volume in 0.1 s (FEV0.1)/FVC ratio was performed with the same computer-controlled small-animal ventilator connected to a negative pressure reservoir (SCIREQ) [24,25]. Mice were tracheotomized and ventilated as described above. The lungs were inflated to a pressure of 30 cm H_2_O over 1 s and held at this pressure. After 0.2 s, the pinch valve (connected to the ventilator) was closed, and after 0.3 s, the shutter valve (connected to the negative pressure reservoir) was opened for exposure of the lungs to the negative pressure. The negative pressure was held for 1.5 s to ensure complete expiration. The FEV/FVC was determined using FlexiVent software.

### 2.8. Statistical Analysis

All values are expressed as the mean ± S.E.M. A two-way ANOVA followed by the Tukey test or the Student’s *t*-test for unpaired results was used to evaluate differences between three or more groups or between two groups, respectively. Differences were considered to be significant for values of *p* < 0.05.

## 3. Results

### 3.1. Expression of Metallothionein in Lung Tissues

**Figure 1:**
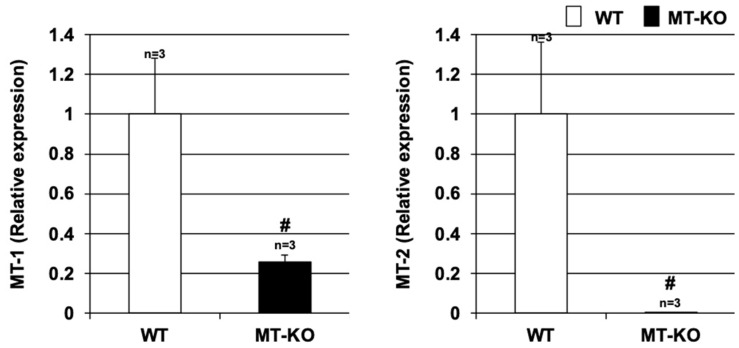
**Expression of metallothionein in lung tissues.** Lung tissues were harvested from wildtype (WT) or MT-1 and -2 gene knockout (MT-KO) mice, and total RNA was extracted from these tissues. The resulting samples were subjected to real-time RT-PCR performed with a specific primer set for each gene. Values were normalized to Gapdh and are expressed relative to the WT. Values are the mean ± S.E.M.; # *p* < 0.05 (#, vs. WT). The experiments that produced the data shown in this figure were performed at least twice.

Lung tissues were harvested from wildtype (WT) mice and MT-KO mice, and the expression of Mt-1 and Mt-2 in the harvested lung tissues was examined by real-time RT-PCR. As shown in Figure 1, the expression of Mt-1 and Mt-2 in the excised lung tissue was significantly lower in the MT-KO mice. Thus, we next examined the effect of metallothionein on elastase-induced lung injury in these two types of mice.

### 3.2. Effect of Metallothionein on PPE-Induced Lung Injury

**Figure 2:**
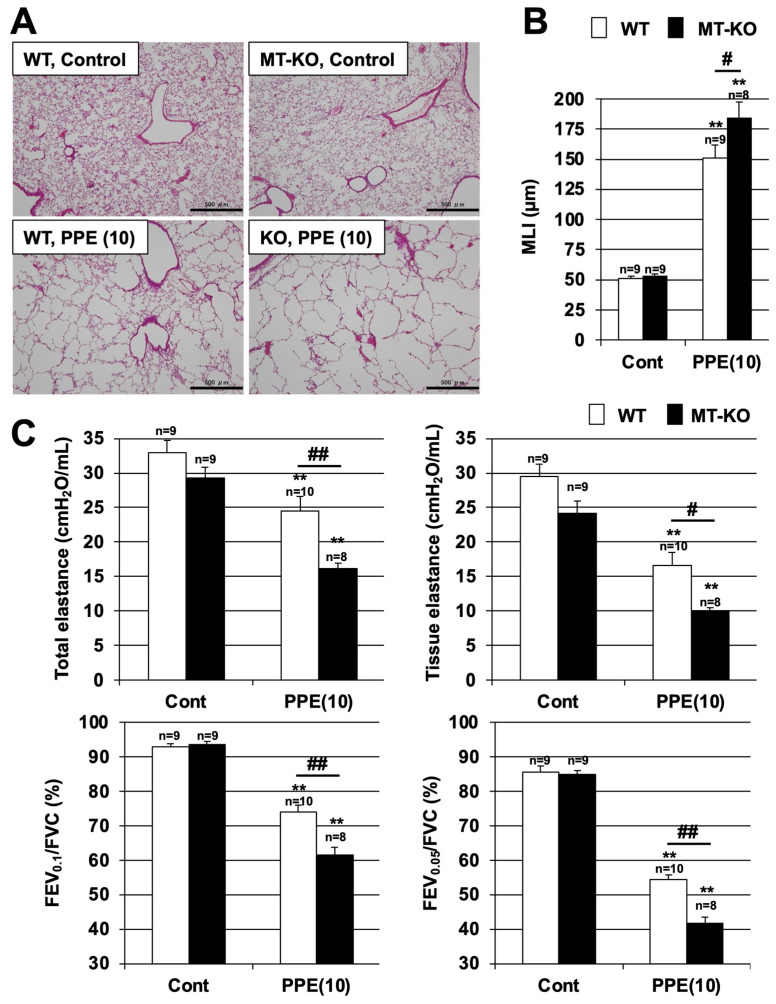
**Effect of metallothionein on PPE-induced pulmonary damage.** (**A**–**C**) Wildtype (WT) and MT-1 and -2 gene knockout (MT-KO) mice were treated with sterile saline (Cont) or PPE (10 U/kg) only once, on day 0. Sections of pulmonary tissue were prepared on day 14 and subjected to histopathological examination (H&E staining) (scale bar, 500 µm) (**A**). Airspace size was estimated by determining the MLI as described in the Materials and Methods (**B**). Total elastance, tissue elastance, FEV_0.05_/FVC, and FEV_0.1_/FVC values were determined on day 14 as described in the Materials and Methods (**C**). Values are the mean ± S.E.M. # *p* < 0.05; ** or ## *p* < 0.01 (**, vs. Control; # or ##, vs. WT). The experiments that produced the data shown in this figure were performed at least twice.

We first examined the effect of metallothionein in the PPE-induced lung injury model, which is one of the animal models of COPD. It is known that PPE administration to mice induces alveolar enlargement and respiratory impairment as evidenced by decreased lung elastance levels and FEV/FVC ratios [10]. Alveolar enlargement was observed in both WT and MT-KO mice after an intratracheal administration of PPE, but this enlargement was more pronounced in the MT-KO mice. We used H&E-stained images to measure the MLIs and found that the MLI was significantly higher in MT-KO mice than in WT mice after PPE administration. In contrast, there was no difference in the MLIs of these mice in the absence of PPE (Figure 2A,B). These results suggest that metallothionein may have a protective effect against alveolar enlargement in COPD.

The FEV_1_/FVC ratio, measured using a spirometer, is the most important indicator for the diagnosis and examination of COPD [2]. In COPD animal models, in addition to decreased FEV/FVC ratios, the levels of elastance, which is an index of lung stiffness, also decrease [10]. As shown in Figure 2C, PPE treatment decreased the levels of total elastance (i.e., elastance from the whole lung, including the bronchi, bronchioles, and alveoli) and tissue elastance (elastance from the alveoli) in both types of mice. Moreover, the TLC, and tissue damping were measured as additional indices, and the results show a significantly accelerated PPE-dependent increase in the TLC and a decrease in tissue damping in MT-KO mice, along with worsened respiratory function in MT-KO mice (Supplementary Figure S2). In addition, the 0.05-s and 0.1-s rates (FEV_0.05_/FVC and FEV_0.1_/FVC ratio, respectively), which are equivalent to the FEV_1_/FVC ratio in humans, were also decreased in both types of mice. The decrease in all indices was more pronounced and statistically significant in the MT-KO mice than in the WT mice. Taken together, the results shown in Figure 2 suggest that metallothionein may have an inhibitory effect on the development of COPD.

### 3.3. Effect of Metallothionein on the PPE-Induced Pulmonary Inflammatory Response and Pulmonary Cell Death

**Figure 3:**
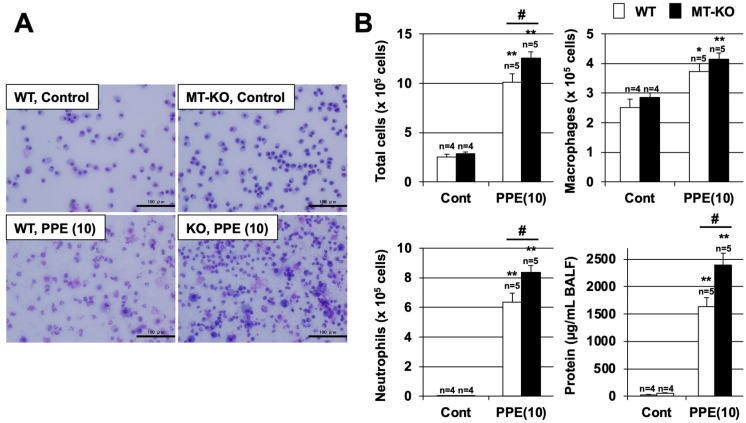
**Effect of metallothionein on PPE-induced acute lung injury.** (**A**,**B**) Wildtype (WT) and MT-1 and -2 gene knockout (MT-KO) mice were treated with sterile saline (Cont) or PPE (10 U/kg) only once, on day 0. BALF samples were collected 72 h after the intratracheal administration of saline or PPE. (**A**) BALF cells were deposited onto slides by using a Cytospin^®^ 4 cytocentrifuge then stained with Diff-Quik reagents and visualized via light microscopy (scale bar, 100 µm). (**B**) The numbers of total cells, macrophages, and neutrophils were determined. The amount of protein present in the BALF was determined by the Bradford method. Values are the mean ± S.E.M. * or # *p* < 0.05; ** *p* < 0.01 (* or **, vs. Control; #, vs. WT). The experiments that produced the data shown in this figure were performed at least twice.

We next compared the increases in the numbers of inflammatory cells in BALF at 3 days after exposure to PPE in WT and MT-KO mice (Figure 3). Under these experimental conditions, the number of total inflammatory cells in BALF increased in a PPE-dependent manner in both WT and MT-KO mice, but the number of total inflammatory cells after PPE treatment was significantly higher in MT-KO mice than in WT mice. In addition, there was no difference in the number of macrophages after PPE administration in either mouse type, but the number of neutrophils involved in acute inflammation was significantly higher in the PPE-exposed MT-KO mice compared with the WT mice. The protein concentration in BALF is an indicator of lung injury and edema. As shown in Figure 3B, treatment with PPE increased the BALF protein concentration in both types of mice, but this increase was more pronounced in the MT-KO mice.

**Figure 4:**
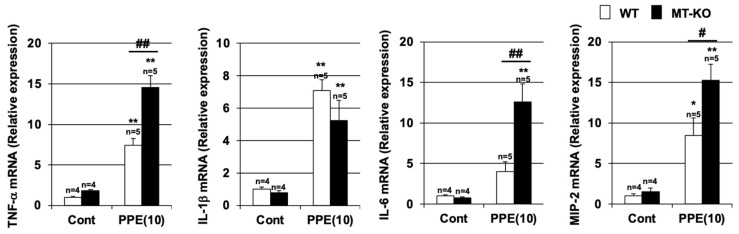
**Effect of metallothionein on PPE-induced pulmonary inflammatory response.** Wildtype (WT) and MT-1 and -2 gene knockout (MT-KO) mice were treated with sterile saline (Cont) or PPE (10 U/kg) only once, on day 0. Total RNA was extracted from the lungs 72 h after PPE administration and subjected to real-time RT-PCR using a specific primer set for each gene. Values were normalized to Gapdh and are expressed relative to the Control. Values are the mean ± S.E.M. * *p* < 0.05; ** or ## *p* < 0.01 (* or **, vs. Control; # or ##, vs. WT). The experiments that produced the data shown in this figure were performed at least twice.

In PPE-induced lung injury models and COPD patients, it has been reported that inflammatory cytokine production is induced in the lungs, which is involved in the promotion of inflammatory responses in the lungs. Thus, we monitored the expression of pro-inflammatory cytokines by using real-time RT-PCR. As shown in Figure 4, the mRNA expressions of tumor necrosis factor-α (TNF-α), interleukin (IL)-1β, IL-6, and macrophage inflammatory protein 2 (MIP2) were induced by PPE treatment. The levels of TNF-α, IL-6, and MIP2 mRNA expression after PPE treatment were higher in MT-KO mice than in WT mice. In contrast, the levels of IL-1β mRNA expression after PPE treatment in both types of mice were similar. Together, the data shown in Figure 3 and Figure 4 suggest that metallothionein may have an inhibitory effect on PPE-dependent inflammatory responses.

**Figure 5:**
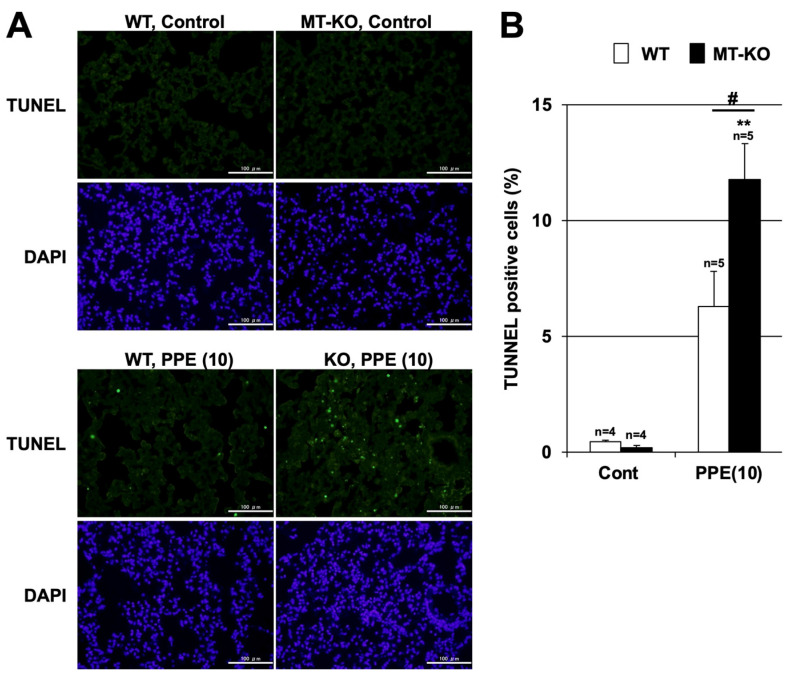
**Effect of metallothionein on PPE-induced pulmonary cell death.** (**A**,**B**) Wildtype (WT) and MT-1 and -2 gene knockout (MT-KO) mice were treated with sterile saline (Cont) or PPE (10 U/kg) only once, on day 0. Sections of pulmonary tissue were prepared 72 h after PPE administration. (**A**) Sections were subjected to terminal deoxynucleotidyl transferase biotin-dUTP nick end labeling (TUNEL) assay and 4,6-diamidino-2-phenylindole (DAPI) staining (scale bar, 100 µm). (**B**) The numbers of TUNEL-positive cells were counted. Values are the mean ± S.E.M. # *p* < 0.05; ** *p* < 0.01 (**, vs. Control; #, vs. WT). The experiments that produced the data shown in this figure were performed at least twice.

Pulmonary cell apoptosis also plays an important role in the pathogenesis of COPD and PPE-induced pulmonary emphysema. Thus, we analyzed cell death in the lungs of MT-KO and WT mice after PPE administration using the TUNEL assay. While few TUNEL-positive cells (i.e., those that succumbed to cell death) were observed in the PPE-untreated groups of mice, the number of TUNEL-positive cells after PPE administration trended higher in WT mice and increased significantly in MT-KO mice (Figure 5). These results suggest that metallothionein may also have an inhibitory effect on PPE-dependent pulmonary cell death. Therefore, we speculate that metallothionein suppresses PPE-induced pulmonary emphysema through its anti-inflammatory and cell death-inhibitory effects.

### 3.4. Effect of Metallothionein on PPE-Induced ROS Production

**Figure 6:**
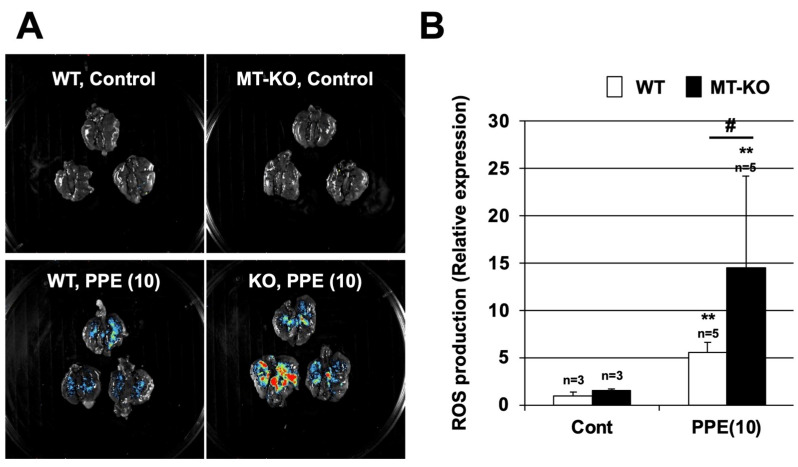
**Effect of metallothionein on PPE-induced ROS production.** (**A**,**B**) Wildtype (WT) and MT-1 and -2 gene knockout (MT-KO) mice were treated with sterile saline (Cont) or PPE (10 U/kg) only once, on day 0. Luminescent probe (L-012, 75 mg/kg) was administered 24 h after the PPE administration. Isolated lungs were imaged using a FUSION chemiluminescence imaging system (**A**). The summed pixel intensity of the ROS signal was determined using standard software for FUSION (**B**). Values are the mean ± S.E.M. # *p* < 0.05; ** *p* < 0.01 (**, vs. Control; #, vs. WT). The experiments that produced the data shown in this figure were performed at least twice.

ROS are involved in the onset and progression of various lung injuries, such as acute respiratory distress syndrome (ARDS) and COPD [26,27]. Because metallothioneins have ROS scavenging activity [13], we used the FUSION in vivo imaging system to monitor ROS levels in our model. Locations with high levels of ROS are indicated by color (highest to lowest levels shown in red, yellow, green, and blue, respectively) in Figure 6. The intratracheal administration of PPE increased ROS production in the lungs of both types of mice (Figure 6). The lungs of MT-KO mice produced more ROS in response to PPE administration compared with those of WT mice. In contrast, there was almost no ROS production in the control-treated WT or MT-KO mice. A quantitative analysis using the standard software provided with FUSION showed that PPE-dependent ROS production was significantly enhanced in the MT-KO mice as compared with WT mice (Figure 6B).

**Figure 7:**
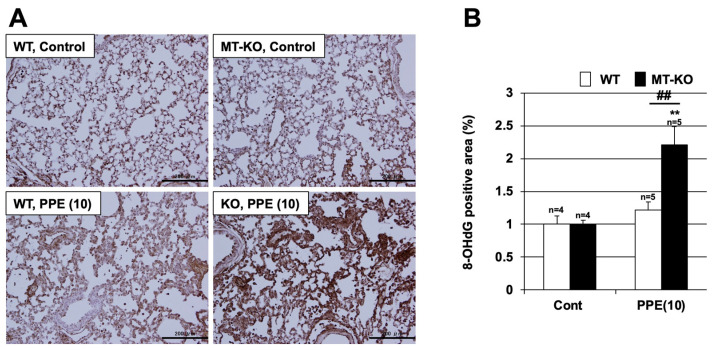
**Effect of metallothionein on PPE-induced oxidative stress.** (**A**,**B**) Wildtype (WT) and MT-1 and -2 gene knockout (MT-KO) mice were treated with sterile saline (Cont) or PPE (10 U/kg) only once, on day 0. (**A**) Sections of pulmonary tissue were prepared 72 h after PPE administration and subjected to 8-hydroxydeoxyguanosine (8-OHdG) staining (scale bar = 200 µm). (**B**) The 8-OHdG-positive area was measured. Values are the mean ± S.E.M. ** or ## *p* < 0.01 (**, vs. Control; ##, vs. WT). The experiments that produced the data shown in this figure were performed at least twice.

To identify oxidative stress in another way, specific antibodies against 8-OHdG (a marker of oxidative damage to DNA) were used. As shown in Figure 7, there was a marked increase in the 8-OHdG-positive area, i.e., an increase in the level of oxidative stress, in the lung tissue of MT-KO mice treated with PPE. In contrast, only a slight increase in the 8-OHdG-positive area was observed in the lung tissue of WT mice treated with PPE.

Finally, to investigate the involvement of oxidative stress in the progression of PPE-induced pulmonary emphysema, we analyzed the ROS production 7 days after PPE administration by using an in vivo imaging system. As shown in Supplementary Figure S3, both types of mice produced less ROS at 7 days after PPE administration than they did at 24 h after PPE administration. However, the level of ROS production at 7 days after PPE administration in the lung tissue of MT-KO mice was significantly higher than that of WT mice. These results suggest that ROS production is enhanced at both the onset and progression stages of PPE-induced pulmonary emphysema in MT-KO mice, resulting in exacerbation of PPE-induced pulmonary emphysema in MT-KO mice relative to that in WT mice.

## 4. Discussion

**Figure 8:**
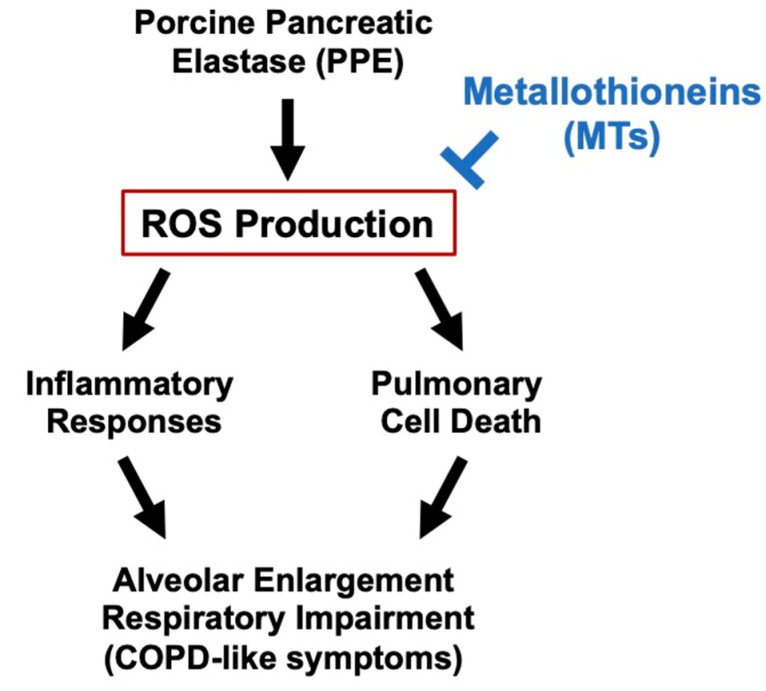
**Mechanism of the inhibitory** **effect of metallothioneins on PPE-induced lung injury.**

In the present study, we found that PPE-dependent lung injury was exacerbated in MT-KO mice compared with their WT littermates. By using an in vivo imaging system, we observed that PPE-dependent ROS production was also significantly enhanced in MT-KO mice. These results suggest that metallothionein may act as an inhibitor against elastase-induced lung injury by suppressing ROS production, which is one of the causes of COPD (Figure 8). Previous analyses using metallothionein-deficient mice have shown that metallothionein is effective against various diseases, including some respiratory diseases. In contrast, there have been few studies on the efficacy of metallothionein against COPD, so it is notable that the present study using MT-KO mice provides evidence regarding the efficacy of metallothionein against COPD.

As described above, metallothionein is known to inhibit the ROS production caused by various stresses [13]. We therefore focused our analysis on the antioxidant effect of metallothionein as a protective mechanism against lung injury, and we demonstrated for the first time, using an in vivo imaging system, that PPE-dependent ROS production is enhanced in MT-KO mice. This work is the first to demonstrate visually that metallothionein inhibits PPE-dependent oxidative stress. Notably, ROS are not only a short-term exacerbating factor of COPD, e.g., following exposure to stress such as CS, but also a cause of chronic COPD exacerbation through their involvement in chronic inflammation and fibrosis [28]. In this study, ROS production was significantly increased in MT-KO mice at not only 24 h but also 7 days after PPE exposure. This increase in ROS levels at 7 days after PPE exposure may be related to chronic inflammation and fibrosis; the details of this mechanism should be investigated in future work. Such research will lead to a better understanding of the molecular mechanisms by which metallothionein inhibits lung injury.

One particularly interesting finding of this study is that the amounts of total elastance and tissue elastance tended to be lower in MT-KO mice than in WT mice, not only after PPE exposure but also in the untreated state. It has been reported that MT-KO mice have a shorter lifespan because of progressive aging [29,30]. Our findings, in addition to those of previous reports, suggest that the induced lower lung function of MT-KO mice may be one reason for their shorter lifespan relative to WT mice. Moreover, the results of this study also suggest the possibility of using metallothionein as a predictor of COPD onset or exacerbation. To the best of our knowledge, no studies have yet analyzed the correlation between the metallothionein amount in the blood, BALF, or lung tissue and COPD susceptibility. If such an analysis can be conducted, it may be possible not only to apply metallothionein inducers as therapeutic agents but also to use metallothionein as a predictor of the onset or exacerbation of COPD.

In our previous analysis, we found that polaprezinc and zinc acetate, which are approved zinc-containing drugs, suppressed the alveolar epithelial cell injury caused by cadmium, a metal found in cigarette smoke, suggesting that metallothionein inducers such as polaprezinc may be effective against COPD [20]. Considering the results of our previous studies together with the results of the present study, it is conceivable that metallothionein-inducing compounds may have efficacy as COPD therapeutic agents. In particular, our results showing that metallothionein inhibits the injury-induced decrease in FEV/FVC ratio, which is a diagnostic index of COPD in clinical practice, are very significant. However, several issues remain to be addressed prior to the clinical application of metallothionein inducers as a COPD treatment. Specifically, it is essential to evaluate this question using a lung injury model for long-term CS exposure. Future work will need to examine a lung injury model with long-term CS exposure using MT-KO mice and their WT littermates and to analyze the efficacy of compounds that induce metallothionein, such as polaprezinc, on the lung injury model induced by long-term CS exposure.

## 5. Conclusions

In this study, we found that both PPE-dependent lung injuries were exacerbated in MT-KO mice. Furthermore, we observed that PPE-dependent ROS production was also significantly enhanced in MT-KO mice. These results suggest that metallothionein may act as an inhibitor against elastase-induced lung injury by suppressing ROS production. In conclusion, metallothionein protein, or compounds that can induce metallothionein, may be useful in the treatment of COPD.

## Data Availability

The data that support the findings of our study are available from the corresponding author upon reasonable request.

## Supplementary Materials

The following supporting information can be downloaded at: https://www.mdpi.com/article/10.3390/biom12040583/s1, Figure S1: Effect of metallothionein on PPE-induced pulmonary damage; Figure S2: Effect of metallothionein on PPE-induced respiratory dysfunction; Figure S3: Effect of metallothionein on ROS production after 7 days of PPE administration.
