# Supplementary material for: Exacerbation of Elastase-Induced Emphysema via Increased Oxidative Stress in Metallothionein-Knockout Mice
Source: Biomolecules. 2022 Apr 15;12(4):583. doi: 10.3390/biom12040583 (PMC9030156; doi:10.3390/biom12040583)
Supplement: Supplementary file 1 [file biomolecules-12-00583-s001.zip › biomolecules-1592391-supplementary.pdf]

Exacerbation of elastase-induced emphysema via increased oxidative stress in metallothionein-knockout mice

Ken-ichiro Tanaka[1,*], Sachie Shiota[1], Okina Sakakibara[1], Mikako Shimoda[1], Ayaka Takafuji[1], Misaki Takabatake[1], Yoshito Kadota[2], Takashige Kawakami[2], Shinya Suzuki[2] and Masahiro Kawahara[1]

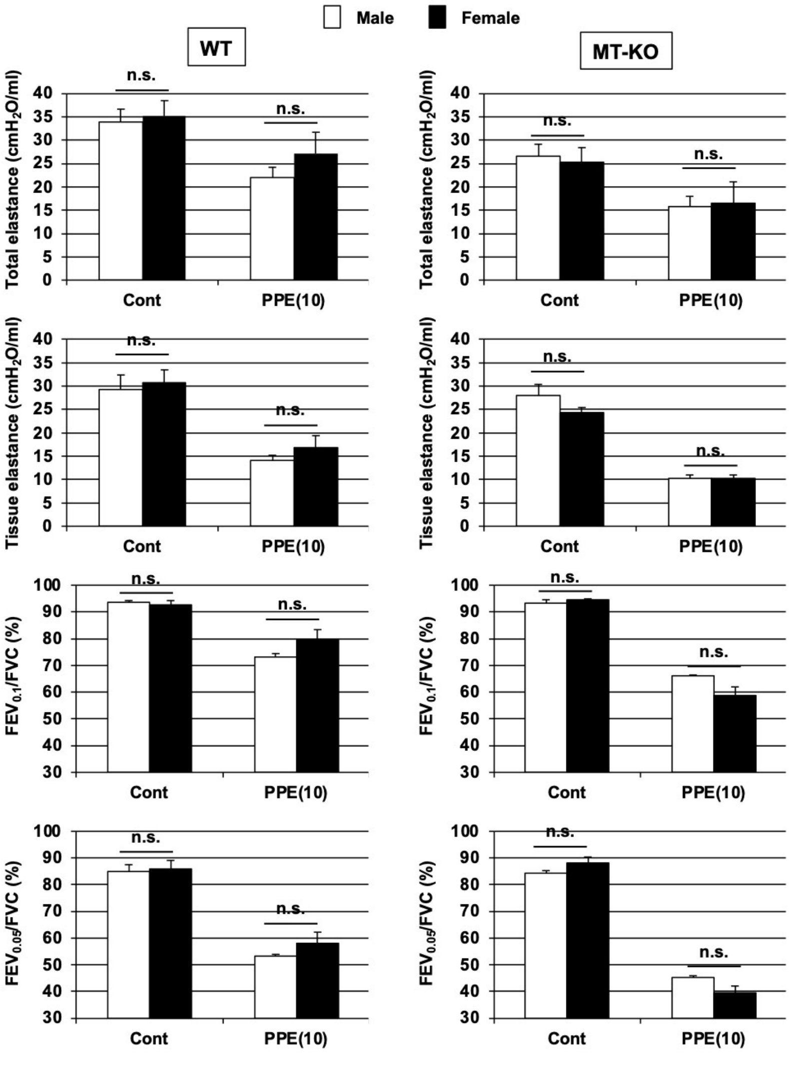

Supplementary Figure S1.
Effect of metallothionein on PPE-induced pulmonary damage.
Wildtype (WT) and MT-1 and -2 gene knockout (MT-KO) mice were treated with sterile saline (Cont) or PPE (10 U/kg) only once, on day 0. Total elastance, tissue elastance, $FEV_{0.05}/FVC$, and $FEV_{0.1}/FVC$ values were determined on day 14 as described in the Materials and Methods. Values are the mean ± S.E.M. n.s.: not significant (Male vs Female). The experiments that produced the data shown in this figure were performed at least twice.

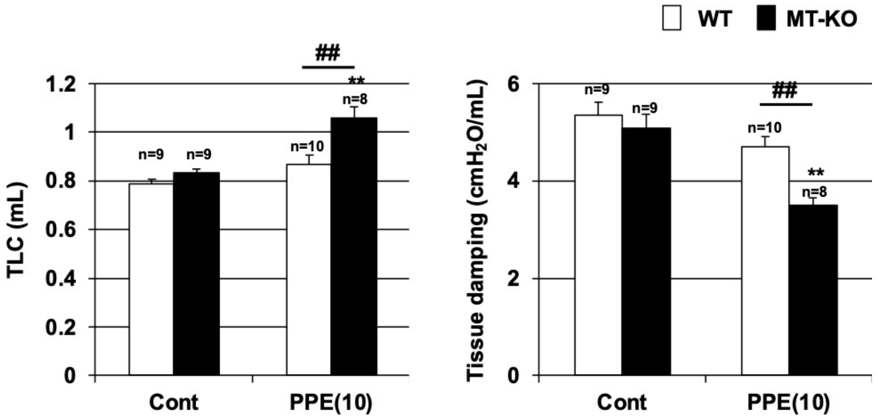

Supplementary Figure S2.
Effect of metallothionein on PPE-induced respiratory dysfunction.
Wildtype (WT) and MT-1 and -2 gene knockout (MT-KO) mice were treated with sterile saline (Cont) or PPE (10 U/kg) only once, on day 0. TLC, and tissue dampling values were determined on day 14 as described in the Materials and Methods (C). Values are the mean ± S.E.M. ** or ##p < 0.01 (**, vs Control; ##, vs WT). The experiments that produced the data shown in this figure were performed at least twice.

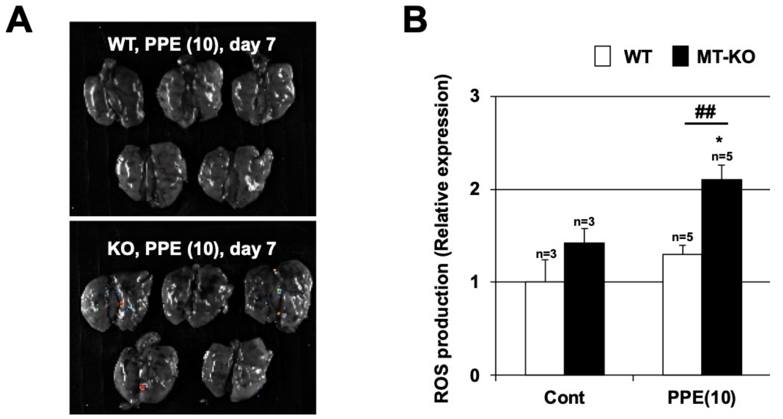

Supplementary Figure S3.
Effect of metallothionein on ROS production after 7 days of PPE administration.
(A, B) Wildtype (WT) and MT-1 and -2 gene knockout (MT-KO) mice were treated with sterile saline (Cont) or PPE (10 U/kg) only once, on day 0. Luminescent probe (L-012, 75 mg/kg) was administered 7 days after the PPE administration. Isolated lungs were imaged using a FUSION chemiluminescence imaging system (A). The summed pixel intensity of the ROS signal was determined using standard software for FUSION. The data for Cont is the same as in Figure 6 (B). Values are the mean ± S.E.M. *p < 0.05; ##p < 0.01 (*, vs Control; ##, vs WT). The experiments that produced the data shown in this figure were performed at least twice.
